# Development of a Rapid-Onset, Acid-Labile Linkage Polyplex-Mixed Micellar System for Anticancer Therapy

**DOI:** 10.3390/polym13111823

**Published:** 2021-05-31

**Authors:** Shiou-Fen Hung, Yu-Han Wen, Lu-Yi Yu, Hsin-Cheng Chiu, Yi-Ting Chiang, Chun-Liang Lo

**Affiliations:** 1 Department of Biomedical Engineering, National Yang Ming Chiao Tung University, No. 155, Sec. 2, Linong St., Beitou Dist., Taipei City 112, Taiwan; sandy49713021@gmail.com (S.-F.H.); qooaz0815@gmail.com (Y.-H.W.); smallfish410048@gmail.com (L.-Y.Y.); 2 Department of Biomedical Engineering and Environmental Sciences, National Tsing Hua University, No. 101, Section 2, Kuang-Fu Road, Hsinchu 300044, Taiwan; hscchiu@mx.nthu.edu.tw; 3 School of Pharmacy, China Medical University, No. 100, Sec. 1, Jingmao Rd., Beitun Dist., Taichung City 406040, Taiwan

**Keywords:** siRNA delivery, acid-labile, mixed micelle, anticancer therapy

## Abstract

In the treatment of cancers, small interfering ribonucleic acids (siRNAs) are delivered into cells to inhibit the oncogenic protein’s expression; however, polyanions, hydrophilicity, and rapid degradations in blood, endosomal or secondary lysosomal degradation hamper clinal applications. In this study, we first synthesized and characterized two copolymers: methoxy poly(ethylene glycol)-b-poly(2-hydroxy methacrylate-ketal-pyridoxal) and methoxy poly(ethylene glycol)-b-poly(methacrylic acid-co-histidine). Afterwards, we assembled two polymers with the focal adhesion kinase (FAK) siRNA, forming polyplex-mixed micelles for the treatment of the human colon cancer cell line HCT116. In terms of the physiological condition, the cationic pyridoxal molecules that were conjugated on the copolymer with ketal bonds could electrostatically attract the siRNA. Additionally, the pyridoxal could form a hydrophobic core together with the hydrophobic deprotonated histidine molecules in the other copolymer and the hydrophilic polyethylene glycol (PEG) shell to protect the siRNA. In an acidic condition, the pyridoxal would be cleaved from the polymers due to the breakage of the ketal bonds and the histidine molecules can simultaneously be protonated, resulting in the endosome/lysosome escape effect. On the basis of our results, the two copolymers were successfully prepared and the pyridoxal derivatives were identified to be able to carry the siRNA and be cleavable by the copolymers in an acidic solution. Polyplex-mixed micelles were prepared, and the micellar structures were identified. The endosome escape behavior was observed using a confocal laser scanning microscopy (CLSM). The FAK expression was therefore reduced, and the cytotoxicity of siRNA toward human colon cancer cells was exhibited, rapidly in 24 h. This exceptional anticancer efficiency suggests the potential of the pH-sensitive polyplex-mixed micellar system in siRNA delivery.

## 1. Introduction

RNA interference (RNAi) therapy, which is to deliver an artificial small interfering RNA (siRNA) to silence the specific expression of genes and proteins [1], has become a promising therapeutic strategy for treating genetic disorders [2,3], including oncogenic cancer [4,5]. Currently, several siRNA-mediated cancer therapies are in the clinical trial stage and have attracted a lot of attention [6,7,8]. However, the nature of siRNAs has led to concerns regarding clinical development [9]. For example, an siRNA can be enzymatically degraded in blood circulation [10]. Additionally, siRNAs are hydrophilic and anionic macromolecules, and hence it is difficult for the siRNA to be internalized into target cells [9,11]. Even when an siRNA is permitted entry into a cell, its ability to escape from endosomes or secondary lysosomes is another concern [9]. In order to overcome these natural obstacles, the siRNA is often packaged into a carrier for the treatment of cancer [9,12,13]. These carriers are required to impede the siRNA from the nuclease during blood circulation, ensure internalization into cancer cells and escape from the endosomes or secondary lysosomes in cells [12,13,14].

So far, many viral and non-viral carriers have been intended for use as an siRNA carrier [9,13,15,16]. Among them, polymer-based siRNA complexes have gained the greatest attention, since the polymers can be easily designed and customized [9]. In fact, some cationic polymers such as polyethylenimine (PEI) and polylysine (PLL) have been introduced into an siRNA delivery system, forming polyplexes [13]. Furthermore, in order to prolong the circulation time in blood and escape from the endosomes or secondary lysosomes, a hydrophilic polymer (polyethylene glycol) (PEG) and histidine molecule have been respectively assembled into polyplexes. The former enabled suitable steric hindrance to prolong the circulation time of the polyplexes [15], whereas for the latter, its imidazole ring could enable protonation as the pH value decreased to 6.5, further leading to increasing the osmolarity and promoting membrane permeability in acidic endosomes or secondary lysosomes, resulting in “proton sponge effects” [16,17]. These effects have successfully led to a significant increase in efficiency for siRNA delivery.

However, the cytotoxicity of cationic polymers is an unignorable drawback in clinical settings [18,19]. It is necessary to exploit polycation materials with low cytotoxicity. As several groups have indicated, the labile bonds in endosomes or secondary lysosomes can efficiently reduce the cytotoxicity [20,21,22,23]; therefore, a labile linkage in endosomes or secondary lysosomes such as an acid labile linker has been introduced into the backbone of the cationic polymers. For example, Bulmus et al. inserted an acid-labile acetal bond in a PEG-macro initiator and prepared a polymer by the reversible-addition fragmentation chain transfer (RAFT) polymerization [24]. Xing et al. prepared ketal containing diacrylate and prepare the acid-labile branched polydiethylenetriamines to form the polyplex with siRNA [25]. Kwon et al. also prepared a poly(ketalized serine) polymer to encapsulate the siRNA [26]. These acetal and ketal bonds, which could be cleavage in acidic milieus, have been therefore suggested to be a promising strategy for an siRNA delivery system.

**Scheme 1:**
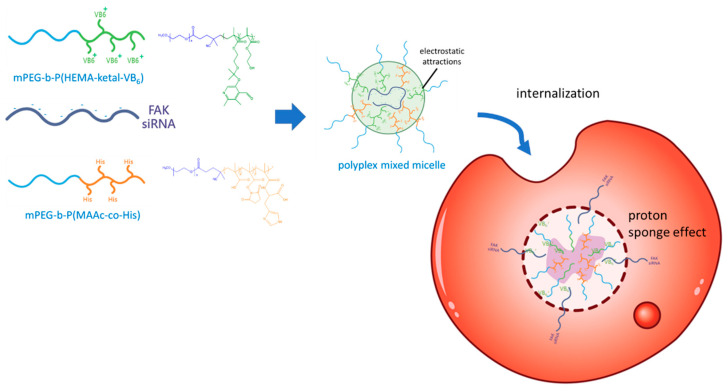
Designs and concepts of the pH-sensitive polyplex-mixed micellar system. The FAK siRNA was assembled with the cationic copolymer, mPEG-b-P(HEMA-ketal-VB_6_), and the histidine molecules appended copolymer, mPEG-b-P(MAAc-co-His). In a physiological condition, the two polymers would form the polyplex-mixed micelles to stably encapsulate the siRNA; while in endosomes or secondary lysosomes, upon a decreasing pH value, cationic vitamin B_6_ derivatives (pyridoxal) are cleaved, and meanwhile, the histidine molecules would be protonated to trigger the proton sponge effects. Finally, the siRNA will release to the cytosol.

In this study, we introduced an acid-labile ketal bond to conjugate a cationic and highly biosafe vitamin B_6_ derivative, pyridoxal into the copolymer, thus forming methoxy poly(ethylene glycol)-b-poly(2-hydroxy methacrylate-ketal-pyridoxal) (mPEG-b-P(HEMA-ketal-VB_6_)). Pyridoxal contained a positively charged pyridine that could be utilized to carry the anionic siRNA in physiological conditions. Because ketal bonds could be cleaved in acidic environments [27,28], the siRNA, as well as the vitamin B_6_ derivatives, were released from the polymers. Moreover, in order to confirm endosomal escape ability and optimize the formation, we further designed and prepared a copolymer, methoxy poly(ethylene glycol)-b-poly(methacrylic acid-co-histidine) (mPEG-b-P(MAAc-co-His)) to carry the histidine molecules. In a physiological environment, the deprotonated histidine molecules are hydrophobic [29]. Therefore, they could be driven close to the pyridine rings of pyridoxal in mPEG-b-P(HEMA-ketal-VB_6_), forming a hydrophobic core and hydrophilic PEG shell. Furthermore, the anionic siRNA could be attracted and encapsulated into the core-shell structure via an electrostatic interaction between siRNA and cationic VB_6_, forming a polyplex-mixed micellar system as shown in Scheme 1. The mixed micellar structures protected the siRNA from degradation during blood transportation. In endosomes or secondary lysosomes, the proton sponge effects were triggered by the histidine residues, and the siRNA could be released into the cytosol. In this study, a 21 base-pair (bp) FAK siRNA was used to treat human colon cancer cells. The FAK proteins have been reported in terms of their relevance to cell survival, growth, and migration [30], in particular for the cancer cells [31]. In other words, the inhibition of FAK proteins with RNAi therapy lead to the cancer-cell death, and anticancer efficiency might be guaranteed. Moreover, polyplex-mixed micelles for FAK siRNA delivery were investigated in terms of anticancer therapy in this study.

## 2. Materials and Methods

### 2.1. Materials

A FAK siRNA was designed by MDBio Inc. (Taipei City, Taiwan) to target the 5′-AACCACCTGGGCCAGTATTAT-3′ (21 base pairs) sequence, and the company also provided the fluorescein isothiocyanate (FAM)-labeled siRNA. The chemical reagents used to synthesize the copolymers, including methoxy poly(ethylene glycol) (Molecular weight: 5000), vitamin B_6_ derivative, pyridoxal hydrochloride, L-histidine, and 2-hydroethyl methacrylate (HEMA), were purchased from MilliporeSigma (Munich, Germany). Additionally, other chemical reagents including *N*-hydroxysuccinimide (NHS ester), 2,2′-dimethoxypropane (DMP), p-toluenesulfonic acid (PTSA) and 4-dimethylaminopyridine (DMAP) were purchased from Alfa Aesar (Ward Hill, MA, USA), whereas the monomer, methacrylic acid (MAAc), was obtained from Acros Organics (Geel, Belgium) and the *N*,*N*′-dicyclohexylcarbodiimide (DCC) was purchased from Fluka, Honeywell International Inc. (Charlotte, NC, USA). The organic solvents, including ethanol and *N*,*N*′-dimethylformamide (DMF), and *N*,*N*-dimethyl acetamide (DMAc) were acquired from TEDIA Company, Inc. (Fairfield, OH, USA). The cell culture medium, McCoy’s 5A medium, was obtained from MilliporeSigma (Munich, Germany) and the MTS cell proliferation assay kits for cytotoxic evaluation were purchased from Promega Corporation (Madison, WI, USA). The fluorescent dye, Lysosensor Blue DND-167, was acquired from Invitrogen, Thermo Fisher Scientific Corporation (Waltham, MA, USA). The regents for the Western blot analysis, including 40% of acrylamide, ammonium persulfate, and *N*,*N*,*N*,*N*-tetramethylethylenedi (TEMED), were obtained from Bio-Rad Laboratories, Inc. (Hercules, CA, USA) and the buffers, including the TRIS buffer, RIPA lysis buffer, and loading buffer, were respectively purchased from Amresce LLC (Fountain Parkway Solon, OH, USA) and Biotools Co., Ltd. (Taipei City, Taiwan). The primary antibodies for Western blot analysis, β-tublin antibody and rabbit anti-PTK2 polyclonal antibody, were, respectively, obtained from EnoGene Biotech Co, Ltd. (New York, NY, USA) and ABclonal Technology (Woburn, MA, USA). The secondary antibodies, including goat anti-rabbit IgG and rabbit anti-mouse IgG, were acquired from Jackson ImmunoResearch Inc. (West Grove, PA, USA).

### 2.2. Preparation and Characterization of mPEG-b-P(HEMA-Ketal-VB_6_)

In order to synthesize the pH-sensitive cationic copolymer mPEG-b-P(HEMA-ketal-VB_6_), the copolymer methoxy poly(ethylene glycol)-block-poly(hydroxyethyl methacrylate) (mPEG-b-PHEMA) was first prepared. Thereafter, the vitamin B_6_ derivative, pyridoxal hydrochloride, was conjugated on the copolymer mPEG-b-PHEMA via acid-labile ketal linkages, forming the copolymer mPEG-b-P(HEMA-ketal-VB_6_).

The preparation of the mPEG-b-PHEMA was carried out following our previous reports [32,33]. In brief, the macroinitiator, methoxy poly(ethylene glycol)2-4,4′, -azobis94-cyanovaleric acid) (mPEG2-ABCPA), was first prepared. The macroinitiator (1 mmole) was dissolved into ethanol under nitrogen, and simultaneously 2-hydroxyethyl methacrylate (40 mmole) was added. After homogenous blending, the solution was heated until 70 °C for 24 h. Afterwards, the mPEG-b-P(HEMA) polymer was precipitated by the iced ether. After drying in a vacuum oven, the product was characterized using a hydrogen nuclear magnetic resonance (^1^H-NMR) (400 mHz, Bruker Avance III 400, Billerica, MA, USA) spectroscopy and Fourier transform infrared spectroscopy (FT-IR) (Affinity-1, Shimadzu, Kyoto, Japan).

The mPEG-b-PHEMA was further conjugated with pyridoxal molecules via ketal bonds to prepare the mPEG-b-P(HEMA-ketal-VB_6_) copolymer. The preparation was carried out as follows: First, the 2′2-dimethoxypropane (DMP) (1.4 mmole), the pyridoxal hydrochloride (2 mmole) and the p-toluenesulfonic acid (0.003 mmole) were placed in a round flask and dissolved into anhydrous *N*,*N*-dimethylacetamide (DMAc). Afterwards, the solution was heated to 80 °C in an oil bath. Meanwhile, the synthesized copolymer, mPEG-b-PHEMA (0.025 mmole), was placed into another round flask and dissolved into the anhydrous DMAc. At 3, 6, 12 and 24 h post-reaction, the mPEG-b-PHEMA solution was added into the DMP and pyridoxal solutions at 80 °C. The mixture was further reacted for 3 h at 80 °C. Afterwards, the solution was cooled down to room temperature and purified by iced ether. The precipitates in the ether were collected and dried in a vacuum over. The chemical structures of the dried powders were characterized with ^1^H-NMR and FT-IR. The optimization of the reaction periods was determined by the conjugation rates, calculated from ^1^H-NMR.

### 2.3. pH-Responsiveness of the Copolymer mPEG-b-P(HEMA-Ketal-VB_6_)

The copolymer mPEG-b-P(HEMA-ketal-VB_6_) (10 mg) was dissolved in pH 7.4 and pH 5.0 phosphate buffer saline (PBS) solutions. The solutions (1 mL) were placed into dialysis bags (M.W.C.O. 6–8 k). Afterwards, the dialysis bags were independently immersed in 3 mL of pH 7.4 or pH 5.0 PBS and incubated at 37 °C. At 0, 1, 2, 3, 6 and 24 h post-incubation, the PBS was collected and pyridoxal concentrations were detected using an ultraviolet-visible light spectrometer (UV-vis spectrometer) (Lambda 35, PerkinElmer, Inc., Waltham, MA, USA). The absorbance at a 410 nm wavelength was recorded.

Moreover, the copolymer mPEG-b-P(HEMA-ketal-VB6), which was dissolved in pH 7.4 and pH 5.0 PBS and incubated at 37 °C for 24 h, was also measured using a gel permeation chromatography (GPC) system equipped with a refractive index (RI) detector (Shimadzu Corporation, Kyoto, Japan).

### 2.4. Preparation and Characterization of mPEG-b-P(MAAc-co-His)

In order to prepare the mPEG-b-P(MAAc-co-His) copolymer, the copolymer mPEG-b-PMAAc was prepared in advance. The preparation of the mPEG-b-PMAAc was also carried out according to our previous report [32,33]. Briefly, the macroinitiator, mPEG2-ABCPA (1 mmole), was weighed and placed into a two-neck bottle. Under nitrogen, the organic solvent, ethanol, was added to dissolve the macroinitiators. Afterwards, the monomer, methacrylic acid (40 mmole), was added in the solution under nitrogen. After homogeneously blending, the solution was reacted at 70 °C. Twenty-four hours later, the solution was cooled down to room temperature and gradually dropped into iced ether for purification. The precipitates in the ether were collected and dried in a vacuum oven. The dried product was identified using ^1^H-NMR and FT-IR.

Thereafter, the mPEG-b-PMAAc copolymer was partially modified with N-hydroxysuccinimide (NHS ester) via a coupling reaction. The preparation was carried out as follows: The copolymer mPEG-b-PMAAc (0.1 mmole) was placed into a round bottom flask with NHS ester (4.5 mmole), 4-dimethylaminopyridine (DMAP) (0.003 mmole), and dicyclohexylcarbodiimide (DCC) (9 mmole). Afterwards, the anhydrous organic solvent dimethylformamide (DMF) was added under nitrogen to dissolve the compounds. The solution was reacted at 25 °C for 24 h. To terminate the reaction, acetic acid (3 mmole) was dropped into the solution. The product, methoxy poly(ethylene glycol)-block-poly(methacrylic acid-co-NHS ester) (mPEG-b-P(MAAc-co-NHS ester)), was obtained via precipitation in the iced ether. After drying in a vacuum oven, the product was characterized using ^1^H-NMR and FT-IR.

The synthesized polymer, mPEG-b-P(MAAc-co-NHS ester) was further reacted with the L-histidine molecules. The mPEG-b-P(MAAc-co-NHS ester) copolymer (0.1 mmole) and the L-histidine (3 mmole) was dissolved in DMF and deionized water, respectively. The two solutions were mixed together afterwards and reacted at 25 °C for 48 h. The polymer solution was further purified via dialysis against deionized water and freeze-dried. The powder was identified using ^1^H-NMR and FT-IR.

### 2.5. Polyplex Preparation and Characterization

The various moles of the mPEG-b-P(HEMA-ketal-VB_6_) were dissolved in 2 mL of dimethyl sulfoxide (DMSO) and mixed with 1 mL of PBS. Afterwards, various concentrations of the polymer solutions were blended with 0.5 mL of the FAK siRNA solution (1 µg/mL) and incubated at 25 °C. At 3–4, 8–9, and 24 h post-incubation, the polyplex solutions were independently placed into a dialysis bag (M.W.C.O. 6–8 k) and dialyzed against deionized water. After dialysis, the solutions were placed in an ultracentrifuge filter tube (M.W.C.O. 10 k) and the volumes of the solutions were concentrated until reaching 3.5 mL. The polyplex samples were analyzed with agarose gel electrophoresis and measured within the UV-vis spectrum. Additionally, the particle sizes of the polyplexes were measured with dynamic light scattering (DLS) (Zetasizer 3000HSA, Malvern Panalytical, Worcestershire, UK). The particle sizes were analyzed using CONTIN method. The morphologies of the polyplexes at pH 7.4 and pH 5.0 were observed using transmission electron microscopy (TEM) (JEM-2000 EXII, JEOL Ltd., Tokyo, Japan) after staining with the 2% uranyl acetate.

### 2.6. Polyplex-Mixed Micelle Preparation and Characterization

The copolymer mPEG-b-P(HEMA-ketal-VB_6_) (1 mmole) was first dissolved in 2 mL of DMSO, and meanwhile, various concentrations of the copolymer, mPEG-b-P(MAAc-co-His) (2, 1 and 0.5 mmole), were independently dissolved into 1 mL of PBS. Afterwards, the two polymer solutions were mixed together, and the siRNA solution was further added into the polymer solution. After incubation at 25 °C for 3–4 and 8–9 h, the solution was placed into dialysis bags and dialyzed against deionized water for 24 h. The polyplex-mixed micelle solution was thereafter placed into an ultracentrifuge filter tube (M.W.C.O. 10 k) and concentrated to 3.5 mL under 1500 rpm for 15 min at 4 °C. The polyplex-mixed micelles were analyzed with agarose gel electrophoresis. Additionally, their particle sizes were measured with DLS under analysis with CONTIN method and their morphologies was observed using TEM with 2% uranyl acetate staining.

### 2.7. Internalization

Fluorescent dye 5′ 6-fluorescein (FAM)-labeled siRNA was prepared into the polyplex-mixed micelles following our abovementioned methods. Afterwards, the polyplex-mixed micelles were stored at 4 °C for the in vitro tests. To observe the endocytic behaviors of the polyplex-mixed micelles in cancer cells, human colon cancer cells HCT116 (1 × 10^5^ cells) were seeded on the slides and incubated at 37 °C with a 5% CO_2_ supply. As the cells were attached, HCT116 cells were treated with polyplex-mixed micelles. After incubation for 1 and 6 h, the excess polyplex-mixed micelles were removed and the cells were washed with PBS thrice. Afterwards, the cells were treated with 1 µM of Lysosensor Blue DND-167 and incubated at 37 °C in darkness. At 2 h post-incubation, excess fluorescent dye was removed and the HCT116 cells were washed. The cells were further fixed with 4% paraformaldehyde for 30 min. After fixation, the cells were washed with PBS thrice and mounted with glycerol. The slides were stored at 4 °C until observation. The internalization and the fluorescence of the cells were observed using a confocal laser scanning microscope (CLSM) (Olympus FV1000, Olympus Corporation, Tokyo, Japan). The fluorescence of polyplex-mixed micelles was detected using an excitation wavelength of 488 nm and emission wavelength of 520 nm. The fluorescence of the endosomes or secondary lysosomes and staining with Lysosensor Blue DND-167 was detected using an excitation wavelength of 405 nm and emission wavelength of 425 nm.

### 2.8. Cytotoxicity Evaluation

Cytotoxicity toward cancer cells was assessed via an MTS assay. Human colon cancer HCT116 cells (1 × 10^4^ cells) were seeded on each well in a 96-well plate. The cells were treated with various concentrations of polymers, including the mPEG-b-P(HEMA-ketal-VB6) and mPEG-b-P(MAAc-co-His) copolymers (31.3, 62.5, 125.0, 250.0, and 1000.0 μg/mL). Afterwards, the MTS reagent (100 µL) was added into each well and the cells were incubated for 37 °C for 3 h. Cell viability was determined using an enzyme-linked immunosorbent assay (ELISA) reader by detection of the absorbance at the 490 nm wavelength.

Additionally, the cells were also treated with various concentrations of the FAK siRNA, polyplex, and polyplex-mixed micelles (3.1, 6.3, 12.5, 25.0 and 100.0 μg/mL), whereas the concentrations of the polyplexes and polyplex-mixed micelles were adjusted in advance based on their carrying siRNA levels. At 24 h post-incubation, the polymers, siRNA, polyplexes, and polyplex-mixed micelles were removed and the cells were washed with PBS thrice. The cell viability was also determined using MTS assay as aforementioned. The results were statistically analyzed using Student’s *t*-test (Excel 2019) and the significant differences are shown with asterisks (*, *p* < 0.05; **, *p* < 0.01; and ***, *p* < 0.001).

### 2.9. Western Blotting Analysis

The in vitro therapeutic efficacies of siRNA and its polyplexes were evaluated by Western blotting analysis. Human colon cancer HCT116 cells (1 × 10^6^ cells/mL) were seeded on each well in 6-well plates. When the cells were attached onto the plates, the FAK siRNA, polyplexes, and polyplex-mixed micelles were independently co-incubated with the HCT116 cells at 37 °C. At 24 h post-incubation, the excess FAK siRNA, polyplexes, and polyplex-mixed micelles were removed and the HCT116 cells were washed with PBS thrice. The cells were collected and redispensed afterwards in a lysis buffer. After reacting with the lysis buffer on ice for 30 min, the cells were further centrifugated at 4 °C under 12,000× g for 5 min. The supernatants were collected to extract the proteins. The extracted proteins were quantified using a Bradford assay (Bio-Rad protein assay, Bio-Rad Laboratories Inc., Hercules, CA, USA). The extracted proteins were thereafter adjusted to the same concentrations and loaded into 10% SDS-PAGE gels for protein separation and analysis. After transferring the proteins from the gels to a polyvinylidene fluoride (PVDF) membrane, the membrane was blocked for 1 h with 5% milk, and afterwards, the membrane was washed thrice. The membrane was further incubated with the FAK primary antibody (rabbit anti-PTK2 polycolonal antibody) and β-tublin antibody solution at 25 °C for 1 h. After removing the antibody solution, the membrane was treated with the secondary antibody (anti-IgG antibody) and reacted for 1 h at 25 °C. When the reaction was complete, the membrane was washed for 3 h and treated with an enhanced chemiluminescence (ECL) reagent. The membrane was observed using a luminescence/fluorescence imaging system (LAS 4000, Fujifilm Holdings Corporation, Tokyo, Japan).

## 3. Results and Discussions

### 3.1. Synthesis and Characterization of Copolymers mPEG-b-P(HEMA-ketal-VB_6_)

**Scheme 2:**
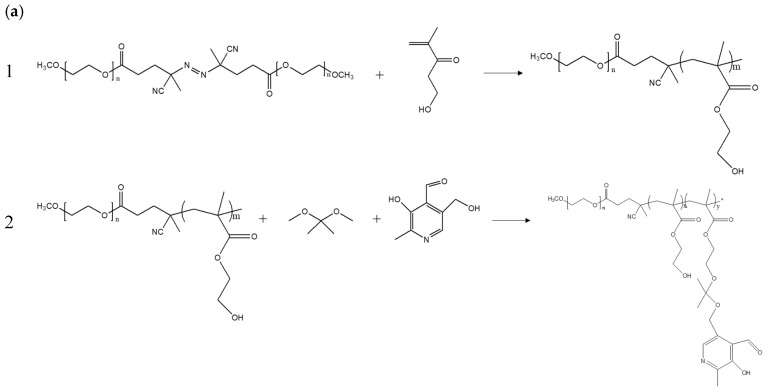

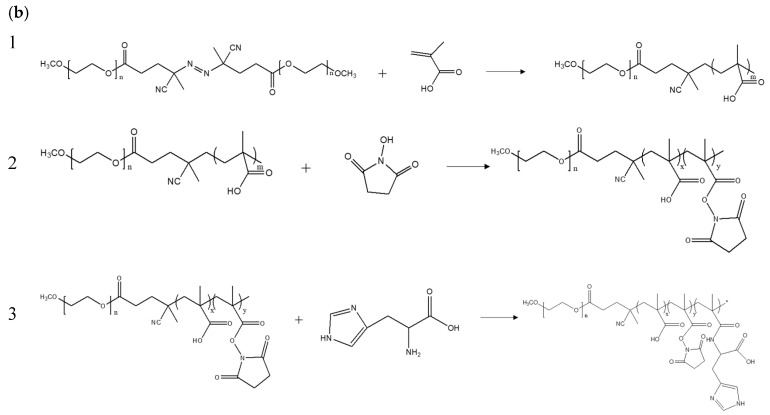
Synthesis of the (**a**) mPEG-b-P(HEMA-ketal-VB_6_) and (**b**) mPEG-b-P(MAAc-co-His) copolymers. (**a**) The synthesis of the copolymer mPEG-b-P(HEMA-ketal-VB_6_) involves two steps: 1. Preparation of the mPEG-b-P(HEMA), using the macroinitator, mPEG2-ABCPA, to conduct the free radical polymerization with the monomer, HEMA; 2. conjugation of the cationic vitamin B_6_ derivative, pyridoxal, using a ketal linker created by 2,2′-dimethoxypropane reagents with catalyst, p-toluenesulfonic acid. (**b**) The synthesis of the mPEG-b-P(MAAc-co-His) copolymers involves 3 steps: 1. preparation of the mPEG-b-P(MAAc) copolymers, using the mPEG2-ABCPA macroinitiator and the MAAc monomers; 2. modification of the mPEG-b-P(MAAc) into mPEG-b-P(MAAc-co-NHS), where the NHS ester was conjugated onto the mPEG-b-P(MAAc) polymers with the ester bonds; and 3. modification of the histidine onto the mPEG-b-P(MAAc-co-NHS) copolymers. The amine groups in histidine were reacted with the NHS groups in the mPEG-b-P(MAAc-co-NHS) copolymers to install the histidine molecules.

In order to encapsulate the anionic siRNA, the cationic polymer mPEG-b-P(HEMA-ketal-VB_6_) was prepared. First, the copolymer mPEG-b-P(HEMA) was synthesized following our previous methods as shown in Scheme 2a [32,33]. In brief, the mPEG-b-PHEMA was prepared from the macroinitiator, methoxy poly(ethylene glycol)2-block-4,4′-azobis(4-cyanovaleric acid) (mPEG2-ABCPA), and the monomer, 2-hydroxyethyl methacrylate (HEMA), with free radical polymerization and precipitation in ether. As Kissel et al. illustrated, the length of the PEG strongly influences the siRNA transfection efficiency and the protection from the nuclease in blood, and a PEG chain molecular weight of 5 kDa could provide sufficient protection, as well as the knockdown of specific proteins [34]. Therefore, in this study, the PEG molecular weight of 5 kDa was chosen and prepared in advance for the macroinitiator, namely, mPEG2-ABCPA. The copolymer was dried and characterized using ^1^H-NMR and FT-IR as respectively shown in Figure S1a,b in Supporting Information. On the basis of the integral areas of the methyl groups in the HEMA and the ethylene groups in PEG in Figure S1a, there were 44 repeating units of mPEG-b-PHEMA. Additionally, Figure S1b indicated that the mPEG-b-PHEMA displayed a strong C–O stretching peak at 1102 cm^−1^ and broad O–H stretching peak at 3425 cm^−1^, respectively, representing the ether bonds in mPEG and the hydroxyl groups in HEMA.

**Figure 1:**
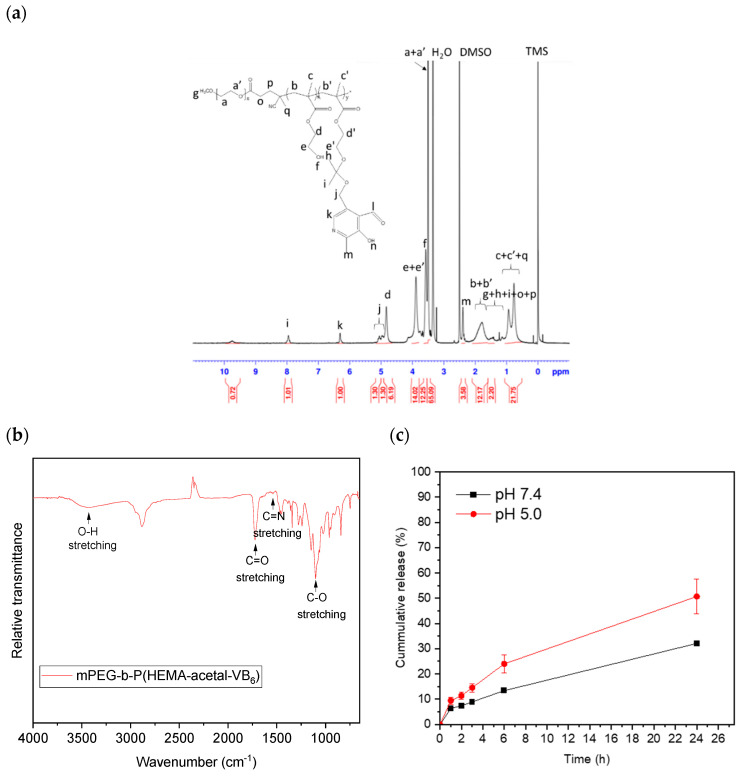
Characterization of the copolymer, mPEG-b-P(HEMA-ketal-VB_6_). (**a**) The ^1^H-NMR spectrum of the mPEG-b-P(HEMA-ketal-VB_6_), after the copolymer was dissolved into DMSO-d_6_. (**b**) The FT-IR spectrum of the mPEG-b-P(HEMA-ketal-VB_6_) copolymers. (**c**) The pyridoxal (vitamin B_6_ derivative) releasing behaviors. The mPEG-b-P(HEMA-ketal-VB_6_) copolymer was dissolved into phosphate buffer saline and placed into dialysis bags. The dialysis bags were further incubated at pH 7.4 and pH 5.0 in PBS and the released pyridoxal was detected in the UV-Vis spectrum.

The synthesized copolymer mPEG-b-PHEMA was reacted with the 2,2-dimethoxypropane and vitamin B6 derivative pyridoxal, forming the copolymer mPEG-b-P(HEMA-ketal-VB_6_), as shown in Scheme 2a, with the catalyst p-toluenesulfonic acid. As suggested by Ozorio et al., the optimal reaction temperature for ketal linkage is 80 °C [35]. As such, we fixed the reaction temperature at 80 °C, and the reaction periods were alternated to optimize the synthesis of the mPEG-b-P(HEMA-ketal-VB_6_) copolymer. After purification with iced ether, the copolymer was dried and characterized with ^1^H-NMR and FT-IR, as shown in Figure 1a,b. The ^1^H-NMR spectrum was utilized to determine the conjugation rate of the copolymers with various reaction periods. As Figure S2 in the Supporting Information indicates, the conjugation rates increased with the increases in the reaction periods before the 15 h incubation period. After the 15 h reaction, the conjugation rate decreased due to the acid-catalyzed hydrolysis. The optimal total reaction period at 80 °C for mPEG-b-P(HEMA-ketal-VB_6_) synthesis was 15 h. The ^1^H-NMR spectrum in Figure 1 indicates that the optimal conjugation rate was approximately 13.2%. The FT-IR spectrum in Figure 1b shows a C=N stretching peak in pyridoxal at 1540 cm^−1^ and also characteristic peaks for mPEG-b-PHEMA, including C–O stretching and a broad O–H stretching peak (Figure S1b in the Supporting Information). The C–O ether stretching of PEG was also found. These results indicate the successful conjugation of VB_6_ onto the copolymer and that the cationic mPEG-b-P(HEMA-ketal-VB_6_) was synthesized.

The ketal linkage of the cationic copolymer was acid-labile, and the pyridoxal could be cleaved from the polymers. Moreover, the pH response of this cationic polymer and the pyridoxal releasing behaviors were identified. Firstly, gel permeation chromatography (GPC) was attempted to introduce and identify the cleavage of the ketal bonds in the cationic mPEG-b-P(HEMA-ketal-VB_6_) copolymer in pH 7.4 and 5.0 at 37 °C; the molecular weight of the polymer is shown in Figure S3 in the Supporting Information. Before incubation, the peak at 8′3”-4” was observed as shown in Figure S3a. After incubation at pH 7.4 for 24 h, the peak did not significantly shift, and a similar GPC spectrum was detected, as shown in Figure S3b. This indicates that the molecular weight of the cationic copolymer did not change. Meanwhile, as the copolymer was incubated at pH 5.0 for 24 h, the GPC spectrum shown in Figure S3c displayed slight differences. As Figure S3c shows, the peak at 8′3”-4” was still detected, but one peak was present at about 13′. These results indicate that the molecular weights of the polymer did not significantly change due to the fact that the conjugation rate of the pyridoxal was only 13%. However, some small molecules were cleaved from the copolymers in pH 5.0 conditions after 24 h incubation, resulting in a peak formation in the GPC spectrum. These GPC results clearly demonstrate that the cationic mPEG-b-P(HEMA-ketal-VB_6_) copolymer can retain its molecular weight in pH 7.4 conditions, while its ketal linkage could be cleaved in acidic conditions; thus, the pyridoxal molecules can be released.

Therefore, we further studied the behaviors of the vitamin B_6_ derivatives released from the polymers. The copolymer mPEG-b-P(HEMA-ketal-VB_6_) was dissolved in pH 7.4 and pH 5.0 PBS and placed in dialysis bags. The dialysis bags were further immersed into pH 7.4 and 5.0 PBS and incubated at 37 °C. At a pre-determined time, the released pyridoxal in PBS was detected as shown in Figure 1c. As Figure 1c shows, when the mPEG-b-P(HEMA-ketal-VB_6_) copolymers were incubated in a pH 7.4 environment for 6 h, little pyridoxal was released; however, a 2-fold increase of released pyridoxal was detected when the polymers were incubated at pH 5.0 for 6 h. After 24 h, over 50% of the vitamin B_6_ derivatives were released from the polyplex-mixed micelles in the pH 5.0 condition, while almost 70% of the vitamin B_6_ derivatives were still preserved in the polyplex-mixed micelles. This release behavior clearly demonstrates the acid-cleavable properties of the cationic mPEG-b-P(HEMA-ketal-VB_6_).

### 3.2. Synthesis and Characterization of Copolymers mPEG-b-P(MAAc-co-His)

In order to provide the proton sponge effect, histidine molecules appending the polymer mPEG-b-P(MAAc-co-His) were prepared. The synthesis was carried out as shown in Scheme 2b. In brief, the mPEG-b-PMAAc was firstly synthesized via free radical polymerization from the macroinitiator mPEG2-ABCPA together with monomer methacrylic acid (MAAc). After purification with iced ether, the polymer was characterized with ^1^H-NMR and FT-IR, as shown in Figure S4a,b in Supporting Information. According to the ^1^H-NMR and FT-IR spectrum, there were approximately 33 repeating units for the copolymers and the successful synthesis of mPEG-b-PMAAc was identified as S-4 in Supporting Information.

Furthermore, the copolymer mPEG-b-PMAAc was partially modified with N-hydroxysuccinimide (NHS) via ester linkage as shown in Scheme 2b. The modified copolymers were identified using ^1^H-NMR and FT-IR spectrum, as Figure S5a,b in Supporting Information shows. On the basis of ^1^H-NMR spectrum, the conjugation rate was 71% and the FT-IR spectrum identified the successful conjugation and synthesis of mPEG-b-P(MAAc-co-NHS ester), as S-5 in Supporting Information illustrated.

**Figure 2:**
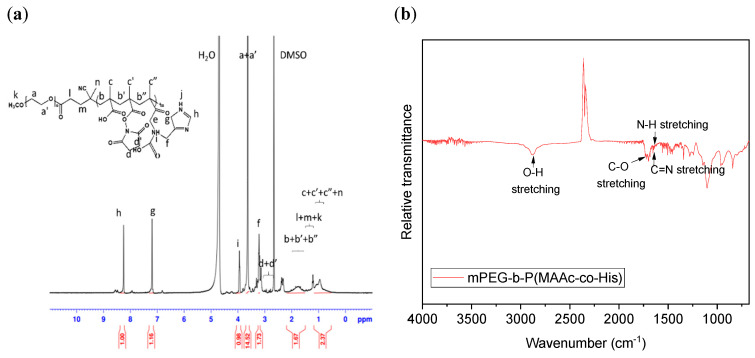
Characterization of the copolymer, mPEG-b-P(MAAc-co-His). (**a**) The ^1^H-NMR spectrum of the mPEG-b-P(MAAc-co-His) copolymer in DMSO-d_6_. (**b**) The FT-IR spectrum of the mPEG-b-P(MAAc-co-His) copolymers.

Afterwards, as Scheme 2b illustrates, the mPEG-b-P(MAAc-co-NHS) reacted with the amine groups in the histidine molecules. The ^1^H-NMR spectra in Figure 2a shows that the peaks at 2.7 ppm were also eliminated, representing that almost all of the NHS ester groups had reacted with histidine molecules. The FT-IR spectrum in Figure 2b further indicates that the N–H stretching and C=N stretching peaks in histidine appeared and that the N–O stretching peaks in NHS groups almost diminished completely, illustrating that the histidine molecules were completely conjugated onto the NHS groups in the polymers, and therefore, the copolymer mPEG-b-P(MAAc-co-His) was successfully prepared. As Hwang et al. suggested, the number of histidine molecules in the polymer significantly affects the proton sponge effects. The histidine molecule count required for endosome escape is over 20 on average. The mPEG-b-P(MAAc-co-His) copolymer conjugated 23 histidine molecules in total, so the endosome escape effect could be achieved [36].

### 3.3. Optimization and Characterization of the Polyplex-Mixed Micelles

The mPEG-b-P(HEMA-ketal-VB_6_) copolymer with positively charged pyridine groups, which is its ability to carry anionic siRNA and form the polyplex, was examined. First, the ratio of the cationic pyridine groups in the copolymer to siRNA phosphate groups, which we defined as N/P ratio, was optimized. Meanwhile, various incubation periods were also in consideration as well. As S-6 and Figure S6a,b in Supporting Information suggested, the polymers and siRNA should be incubated for at least 8–9 h with the ratio of 100:1 such that they could completely form the desired polyplexes. However, the particle sizes of the polyplexes reached to several µm-sized, as the TEM images in Figure S6c indicated. That could be accounted for by the incomplete interactions between the siRNA and the cationic reagents. As Spagnou et al. illustrated, the structures of 21 bp siRNAs are rigid and they have difficulty in terms of condensation in the presence of the cationic reagents, therefore resulting in an undesirably large complex [37]. Particle size plays an important role in internalization. Several studies have indicated that a large polyplex has difficulty in terms of internalization into cancer cells, so it is essential to reduce the particle size [38,39,40]. Additionally, devices for endosome escape are needed. Therefore, we further introduced the polymer mPEG-b-P(MAAc-co-His) to form the polyplex-mixed micelles. With this copolymer, the relative hydrophobic imidazole rings of histidine provided the hydrophobic forces to stabilize the structures and hence reduced the particle sizes [29]. In addition, the histidine residues permitted the proton sponge effects by the protonation of their imidazole rings, causing an influx of chloride ions and leakage of the endosomal/lysosomal membrane via osmotic pressure [9,17]. Herein, the optimization of the polyplex-mixed micelles was investigated. Here, we defined the ratio of the histidine containing polymer and the cationic polymers as H/N ratio. We fixed the ratio of the cationic copolymers and siRNA into 100:1, while various molar ratios of the histidine-containing polymers and cationic polymers were applied to optimize the polyplex-mixed micelles. In addition, the incubation periods for polyplex-mixed micelle formation were to be investigated.

**Figure 3:**
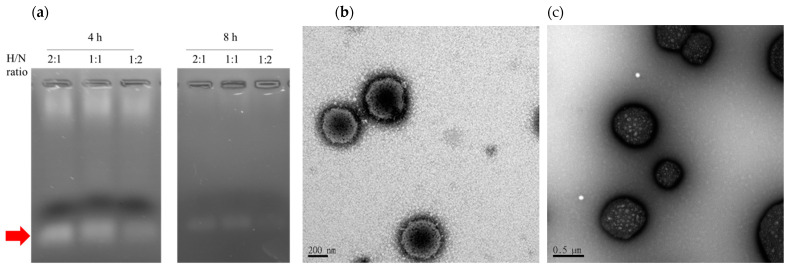
Preparation and characterization of the polyplex-mixed micelles. Various concentrations of the copolymer, mPEG-b-P(MAAc-co-His), were incubated with the cationic copolymer, mPEG-b-P(HEMA-ketal-VB_6_), and siRNA for 3–4 and 8–9 h to form the polyplex-mixed micelles. The polyplex-mixed micelles were analyzed with (**a**) gel electrophoresis. (**b**) The morphologies of the polyplex-mixed micelles were observed using TEM. (**c**) The morphology of the polyplex-mixed micelles after incubation in acidic conditions (pH 5.0). The polyplex-mixed micelles were incubated at 37 °C at pH 5.0 for 24 h; afterward, the morphology was further observed using TEM after staining with uranyl acetate.

The polyplex micelle formation was identified using gel electrophoresis as shown in Figure 3a, which indicates that when the two polymers co-incubated with siRNA for 4 h free siRNA was detected, as indicated by the red arrow. The siRNA and the two equal molar ratios of the polymers were co-incubated for 8 h. Notably, the band representing free siRNA disappeared. The results clearly illustrate that the siRNA was totally encapsulated in the presence of the other constituent polymer mPEG-b-P(MAAc-co-His); however, when the higher level of the mPEG-b-P(MAAc-co-His) was added (H/N ratio is 2:1), the polyplexes were hardly well assembled, as shown in Figure 3a. This arose from the impedance of anionic carboxylate groups in the mPEG-b-P(MAAc-co-His) polymer. The results suggest that in order to completely encapsulate the siRNA we should add the 2 polymers with an equal molar ratio, wherein the ratio of the mPEG-b-P(HEMA-ketal-VB_6_) and siRNA should be 100:1. The particle sizes and the morphologies of the polyplex-mixed micelles were, respectively, identified using DLS and TEM. The average hydrodynamic diameters of the polyplex-mixed micelles were approximately 253.7 nm and the polydispersity index (PDI) was 0.2. Their TEM images (Figure 3b) are in agreement with the particle size results as measured by DLS. Additionally, their micellar core-shell structures could be witnessed in Figure 3b after staining with uranyl acetate. The TEM images clearly and directly demonstrate the formation of mixed micelle polyplexes. Even if the particle sizes were reduced to 250 nm, the gene transfection efficiency was reported to be low for polyplexes with particle sizes over 150 nm [38]. However, as Rejman et al. suggested, internalization still enabled the occurrence in polyplexes over 500 nm, whereas the uptakes decreased upon the alternation of the endocytic pathway [41]. Therefore, the particle sizes were acceptable for siRNA delivery.

Furthermore, the pH responsiveness of the polyplex-mixed micelles was identified by morphological observation. The polyplex-mixed micelles were incubated at 37 °C under pH 5.0 conditions for 24 h. Their morphology was observed using TEM after staining with uranyl acetate. The TEM images, shown in Figure 3c, indicate that, after acidic treatment, the core–shell structures of the polyplex-mixed micelles had destructed and numerous voids were discovered. These phenomena could account for the cleavage of the pyridoxal from the polymers and the protonation of the histidine molecules. The cationic pyridoxal was appended on the polymers with ketal bonds. Under acidic conditions, the pyridoxal could be cleaved from the mPEG-b-P(HEMA-ketal-VB_6_) copolymer, as identified in Figure 1c. Moreover, the imidazole ring in a histidine molecule could be protonated and positively charged when the pH value is lower than 6.5. Therefore, in a pH 5.0 environment, with the detachment of the pyridoxal moieties and positively charged histidine, the polyplex-mixed micelles were destroyed in voids. 

### 3.4. Internalization, Intracellular Releasing Behaviors and Endosome Escape

**Figure 4:**
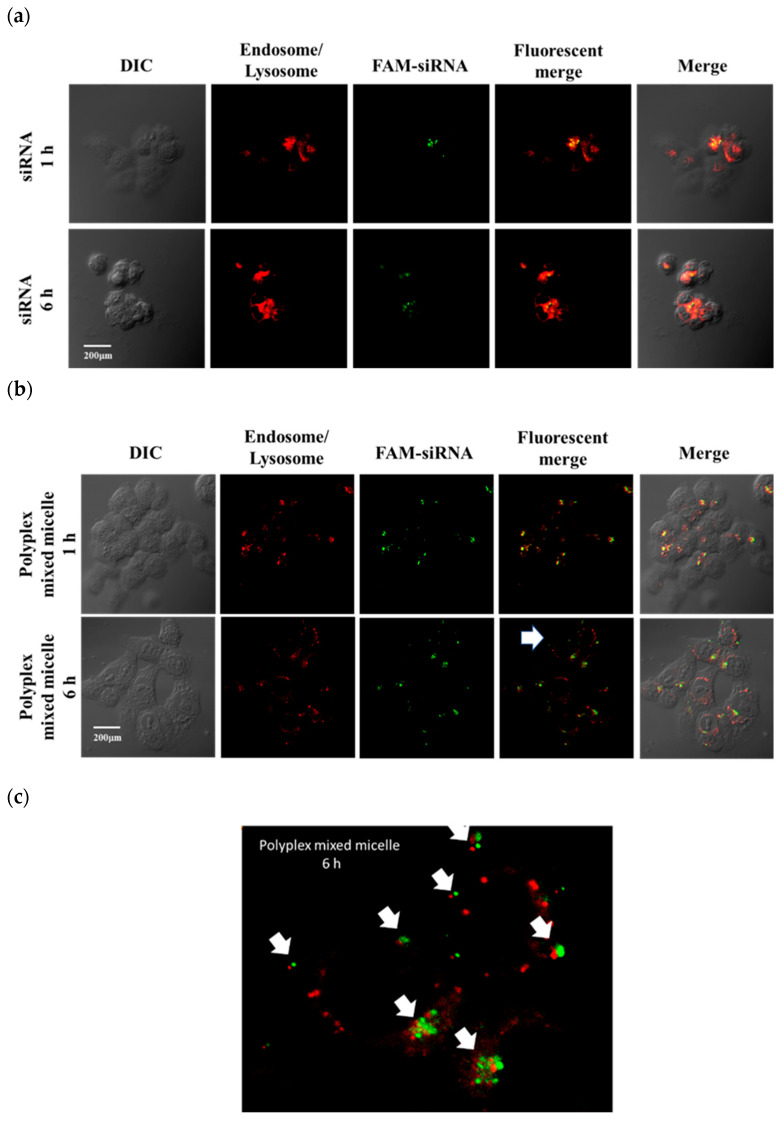
Internalization and endosome escape. The fluorescence dye, (**a**) FAM-labeled FAK siRNA, and (**b**) FAM-labeled polyplex-mixed micelles were respectively incubated with the human colon cancer cells (HCT116) for 1 and 6 h. Afterwards, the endosomes and secondary lysosomes in the cells were stained with a fluorescence dye and the fluorescence of the HCT116 cells was detected using confocal laser scanning microscopy (CLSM), where the FAM fluorescence is present in green and the fluorescence of the endosomes or secondary lysosomes is represented in red. (**c**) At 6 h co-incubation with the human colon cancer cell line, HCT116, the accommodation of FAM-labeled in cells were also observed using CLSM.

To detect and observe the intracellular behaviors of the polyplex-mixed micelles, a fluorescent dye, FAM-labeled siRNA, was utilized to prepare the polyplex-mixed micelles. Afterwards, the FAM-labeled siRNA and polyplex-mixed micelles were respectively incubated with the human colon cancer cell line HCT116 at 37 °C with a 5% CO_2_ supply. At 1 and 6 h post-incubation, the endosome/secondary lysosomes were stained with a fluorescent reagent and the cells were observed using CLSM. As Figure 4a shows, at 1 h post-incubation, green fluorescence was observed inside the human colon cancer cell HCT116 and overlapped with the red fluorescence (present in yellow), indicating that the FAM-labeled siRNA (shown in green) was internalized into human colon cancer cells and accommodated in the endosomes or secondary lysosomes (present in red). At 6 h post-incubation, the green fluorescence was also observed in the endosome or lysosome of the cells, while the fluorescent intensity of the FAM-labeled siRNA subtly decreased, due probably to the degradation of the siRNA in the endosomes or secondary lysosomes. With respect to the polyplex-mixed micelles, the green fluorescence of the FAM-labeled siRNA was observed in the cells after 1 h of incubation, also overlapping with the red fluorescence, showing yellow fluorescence, as Figure 4b shows. The CLSM images demonstrate that the FAM-labeled siRNA was internalized into cancer cells and located in the endosomes/secondary lysosomes. After 6 h of incubation, green fluorescence was still observed, while the fluorescence intensity did not significantly increase during incubation periods. This accounts for the particle sizes of our polyplex-mixed micelles, even though it was noticeable that this green fluorescence was separate from red fluorescence after 6 h of incubation. As Figure 4c shows, most green fluorescence was located near the red fluorescence, indicating that the FAM-labeled siRNA (shown in green) escaped from the endosomes/secondary lysosomes. Due to the high H/N ratios, the escape of endosomes or lysosomes could be attributed to the proton sponge effects of sufficient histidine molecules in the polyplex-mixed micelles [36]. In addition, the acid-cleavable ketal linkage in mPEG-b-P(HEMA-ketal-VB6) might increase the efficiency of the endosome escape. As Li et al. identified, the hydrophobic core of a polyplex micelle results in the reduction of the endosome escape efficiency of the siRNA [42]. During internalization, the hydrophobic core of a polyplex micelle was suggested collapse [9,42]. In our study, the core of the polyplex could be destroyed upon the cleavage of the pyridoxal from the copolymer (as shown in Figure 3c) at low pH. The dissociated core therefore facilitated the siRNA entry into the cytosol.

### 3.5. In Vitro Cytotoxic Evaluation

**Figure 5:**
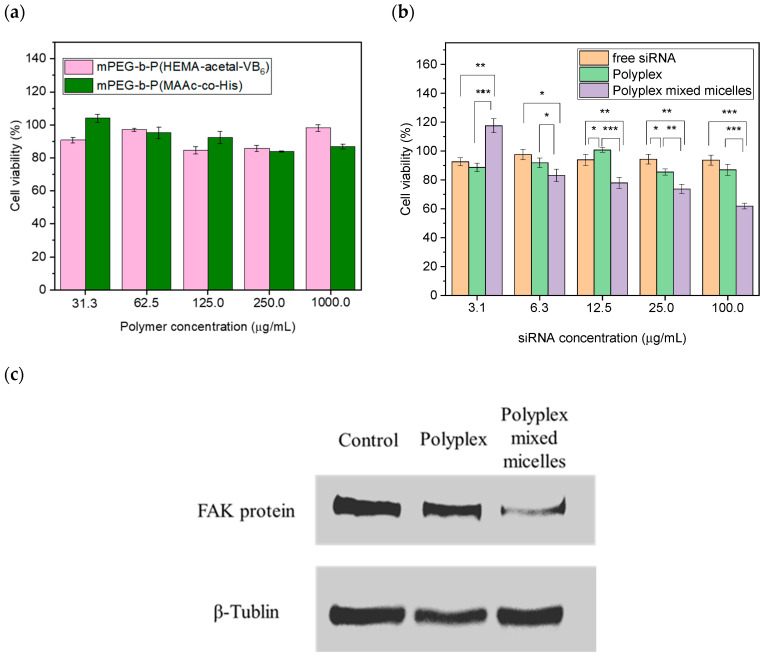
FAK protein expression and cytotoxic evaluation. (**a**) Various concentrations of the constituent copolymers, including mPEG-b-P(HEMA-ketal-VB6) and mPEG-b-P(MAAc-co-His) as incubated with the human colon cancer cells HCT116 for 24 h. (**b**) Various concentrations of the free siRNA, polyplexes, and polyplex-mixed micelles incubated with the human colon cancer cells HCT116 for 24 h. The cell viability was analyzed using an MTS assay. The results were statistically analyzed; significant differences are shown with asterisks (*, *p* < 0.05; **, *p* < 0.01; and ***, *p* < 0.001). (**c**) The FAK siRNA, the polyplexes and the polyplex-mixed micelles were incubated with the human colon cancer cell line HCT116 for 24 h. Afterwards, the proteins in the HCT116 cells were extracted and analyzed via Western blotting.

The therapeutic efficacies and cytotoxicities of the polyplex-mixed micelles were further evaluated for the treatment of the human colon cancer cells HCT116. First, the cytotoxicity of the constituent polymers, including mPEG-b-P(HEMA-ketal-VB_6_) and mPEG-b-P(MAAc-co-His), was examined to ensure biosafety. As Figure 5a shows, both polymers did not cause significant cell death after incubation with the human colon cancer cell line HCT116 for 24 h, even when high concentrations of the polymers were applied. Notably, artificial cationic copolymers have been reported to cause severe cytotoxicity toward a variety of cells; therefore, they were designed to form a polyplex with siRNA under relatively low N/P ratios [38,43]. However, our cationic mPEG-b-P(HEMA-ketal-VB_6_) copolymer displayed very low cytotoxicity, even when high levels of copolymers were utilized to assemble siRNA. This was attributed to the cleavable fashions of the cationic pyridoxal molecules at low pH. As Forrest et al. illustrated, cleavable cationic reagents can efficiently reduce the cytotoxicity of cationic polymers [22]. A cationic moiety, i.e., pyridoxal, could be eliminated from our cationic copolymer mPEG-b-P(HEMA-ketal-VB_6_), leading to a reduction in toxicity toward the cells. It is notable that in previous studies, the researchers installed the acid-labile linkages on the cationic polymer backbone [20,25], while in our study, the cationic moieties were appended on the polymer with acid-labile linkage. The cytotoxic results in Figure 5a demonstrated that our strategy could also efficiently reduce the cytotoxicity.

As Figure 5b shows, when we treated the polyplex-mixed micelles, the cells displayed siRNA concentration-dependent cytotoxicity. When 100 µg/mL of siRNA containing polyplex-mixed micelles was co-cultured with cancer cells for 24 h, the cell viability reduced to 61.9 ± 2.0%, as shown in Figure 5b. It is noticeable that when the cells were treated with 100 µg/mL of the free siRNA, only 10% of cell death occurred as a result. This could reasonably account for the siRNA degradation within endosomes or secondary lysosomes. When the cells were treated with 100 µg/mL for the polyplexes made from the cationic polymer mPEG-b-P(HEMA-ketal-VB_6_), the cell growth was subtly inhibited. After 24 h of incubation the cell viability remained 87.0 ± 3.8%. The low cytotoxicity of the polyplexes toward cancer cells could be attributed to their large particle sizes.

Furthermore, the FAK protein expression was examined using Western blotting. We treated 100 µg/mL of FAK siRNA to the human colon cancer cell HCT116 for 24 h. Afterwards, the expressions of the FAK proteins were analyzed as per Figure 5c. At 24 h post-incubation, the intensity of the FAK protein bands of the cells treated with free siRNA and polyplexes still remained in comparison of the cells without any treatment (control). After 24 h of incubation, as the cells were treated with polyplex-mixed micelles, the intensities of the FAK protein bands appeared to be weak, indicating that the expression of FAK protein was efficiently inhibited when the polyplex-mixed micelles were applied. The result in Figure 5c is in correspondence with that of the cytotoxicity present in Figure 5b. The FAK siRNA in the polyplex-mixed micelles could be internalized into cancer cells and escape from the endosomes/secondary lysosomes (Figure 4b,c). Herein, the siRNA can efficiently inhibit the expression of the FAK proteins, resulting in cancer cell death. However, the siRNA and polyplexes were unable to escape from the endosomes or secondary lysosomes in cancer cells. Finally, the siRNA was gradually degraded in the endosomes or secondary lysosomes during the process of endocytosis. Notably, the naked siRNAs typically need 3 to 7 days to achieve oncogenic knockdown and suppress proteins [9]. However, we could rapidly inhibit the FAK protein suppression due to the acid-labile cationic polymers and proton sponge effect. Our polyplex-mixed micelles enabled the rapid and precise delivery of the siRNA to cytosol, leading to the rapid onset of the RNAi effects. Even though our polyplex-mixed micelles could rapidly release the siRNA and cause the cancer cell inhibition, the cancer cell viability was still higher than the half-maximal inhibitory concentration (IC_50_), as Figure 5b displayed. This may be due to the slow internalization caused by the particle sizes. However, the reduction in particle sizes may be associated with the increase in the rates of the conjugation of the cationic pyridoxal species to the copolymers, and the toxicity can be re-evaluated. This novel polyplex-mixed micellar system revealed a high biosafety and feasibility in siRNA delivery. Therefore, the combination of the FAK siRNA therapy and chemotherapy was suggested to be applied in treatment of the colon cancer in the future.

## 4. Conclusions

In this study, we synthesized two polymers, including an acid-linkage and cationic copolymer, mPEG-b-P(HEMA-ketal-VB_6_), and a copolymer containing histidine molecules, mPEG-b-P(MAAc-co-His). These two polymers were assembled with FAK siRNA to form a polyplex-mixed micellar system. In endosomes or secondary lysosomes, vitamin B_6_ derivatives (pyridoxal) could be cleaved due to the polymer designation, and the histidine molecules presented the proton sponge effect and forced the siRNA to release the cytosol. Therefore, the polymers did not display toxicity, and meanwhile the RNAi effect was rapidly initiated and suppressed the expression of FAK proteins. Eventually, the polyplex-mixed micelles efficiently led to the cytotoxic effects toward the human colon cancer cells HCT116. Vitamin B_6_ derivatives containing pH-responsive polyplex-mixed micelles are promising candidates for rapid onset siRNA vectors for anticancer therapy.

## Data Availability

The data presented in this study are available on request from the corresponding author.

## Supplementary Materials

The following are available online at https://www.mdpi.com/article/10.3390/polym13111823/s1, Figure S1: Preparation and characterization of the mPEG-b-P(HEMA) copolymers, Figure S2: Preparation and characterization of the mPEG-b-P(MAAc) copolymers, Figure S3: Modification and characterization of mPEG-b-P(MAAc-co-NHS) copolymer, Figure S4: Preparation and characterization of the polyplexes.
