# Supplementary material for: Development of a Rapid-Onset, Acid-Labile Linkage Polyplex-Mixed Micellar System for Anticancer Therapy
Source: Polymers (Basel). 2021 May 31;13(11):1823. doi: 10.3390/polym13111823 (PMC8198952; doi:10.3390/polym13111823)
Supplement: Supplementary file 1 [file polymers-13-01823-s001.zip › polymers-1204193-supplementary.pdf]

Supporting Information

Development of a Rapid-Onset, Acid-Labile Linkage Polyplex-Mixed Micellar System for Anticancer Therapy

Shiou-Fen Hung [1], Yu-Han Wen [1], Lu-Yi Yu [1], Hsin-Cheng Chiu [2], Yi-Ting Chiang [3,*] and Chun-Liang Lo [1,*]

1 *Department of Biomedical Engineering, National Yang Ming Chiao Tung University, No.155, Sec. 2, Linong St., Beitou Dist., Taipei City 112, Taiwan; sandy49713021@gmail.com (S.-F.H.); qooaz0815@gmail.com (Y.-H.W.); smallfish410048@gmail.com (L.-Y.Y.); cllo@ym.edu.tw (C.-L.L.)*

2 *Department of Biomedical Engineering and Environmental Sciences, National Tsing Hua University, No. 101, Section 2, Kuang-Fu Road, Hsinchu 300044, Taiwan; hscchiu@mx.nthu.edu.tw*

3 *School of Pharmacy, China Medical University, No. 100, Sec. 1, Jingmao Rd., Beitun Dist., Taichung City 406040, Taiwan; ytchiang@mail.cmu.edu.tw (Y.-T.C.)*

* *Correspondence: ytchiang@mail.cmu.edu.tw (Y.-T.C.); cllo@ym.edu.tw (C.-L.L.);*

Tel.: +886-4-2205-3366 (ext. 5611) (Y.-T.C.); +886-2-2826-7914 (C.-L.L.)

S-1. Preparation and characterization of mPEG-b-P(HEMA) copolymer

Figure S1.

(a)

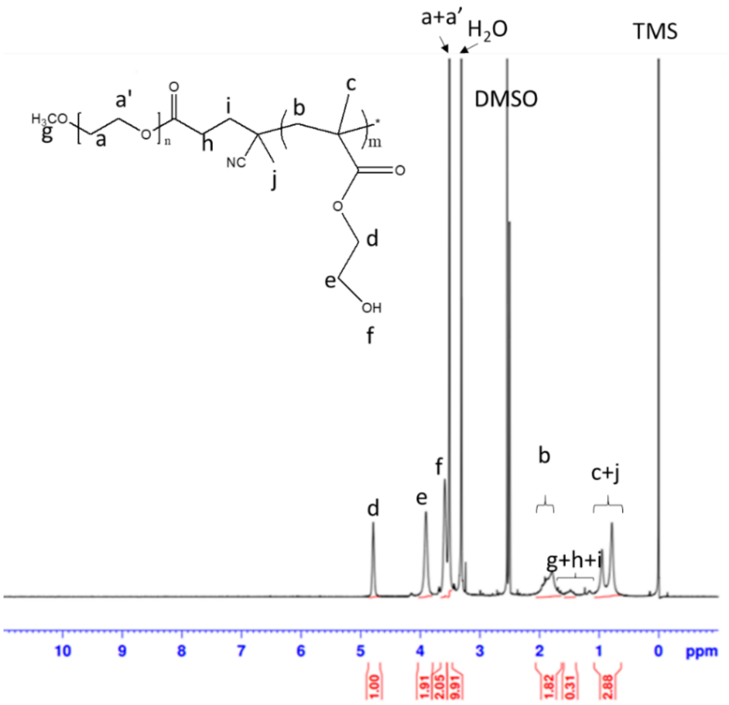

(b)

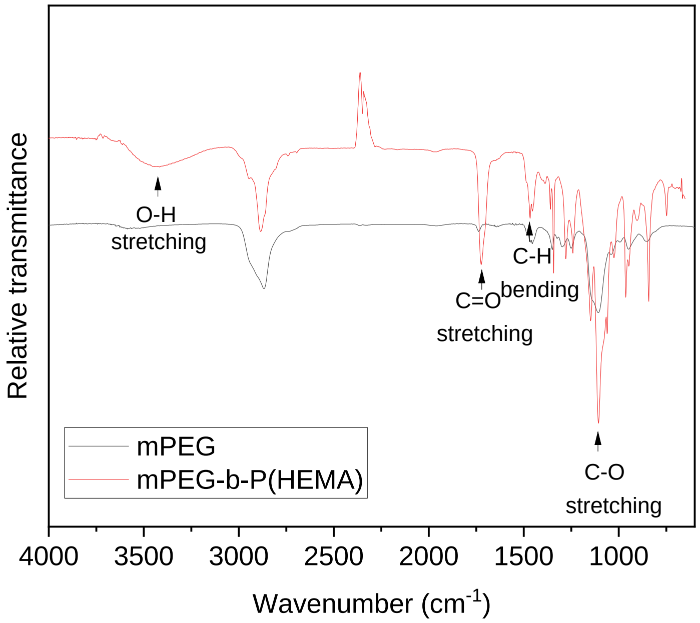

Figure S1. Preparation and characterization of the mPEG-b-P(HEMA) copolymers. (a) The ^{1}H-NMR spectrum of the mPEG-b-P(HEMA) copolymers in DMSO-d_6. (b) The FT-IR spectrum of the mPEG-b-P(HEMA) copolymers.

S-2. Optimization of the reaction periods of mPEG-b-P(HEMA) copolymer

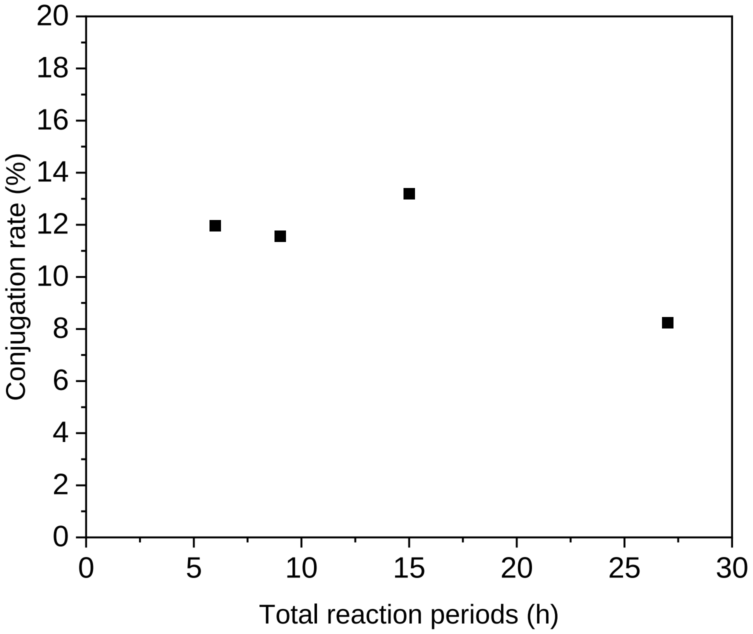

Figure S2. Optimization of the reaction periods of mPEG-b-P(HEMA-ketal-VB_6) copolymer. The 2,2-dimethoxypropane (DMP), the pyridoxal hydrochloride, and the catalyst, p-toluenesulfonic acid (PTSA) were dissolved into anhydrous N,N-dimethylacetamide (DMAc) and reacted at 80°C for 3, 6, 12, and 24 h. Afterwards, the mPEG-b-PHEMA was added and further reacted for 3 h at 80 °C. After precipitation in iced ether, the conjugation rates were determined with ^{1}H-NMR to optimize the reaction conditions.

S-3. Gel permeation chromatography of mPEG-b-P(HEMA-ketal-VB_6) in different pH conditions

(a)

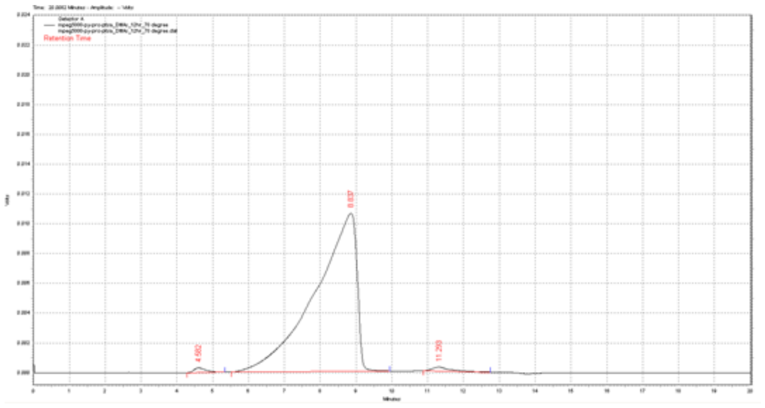

(b)

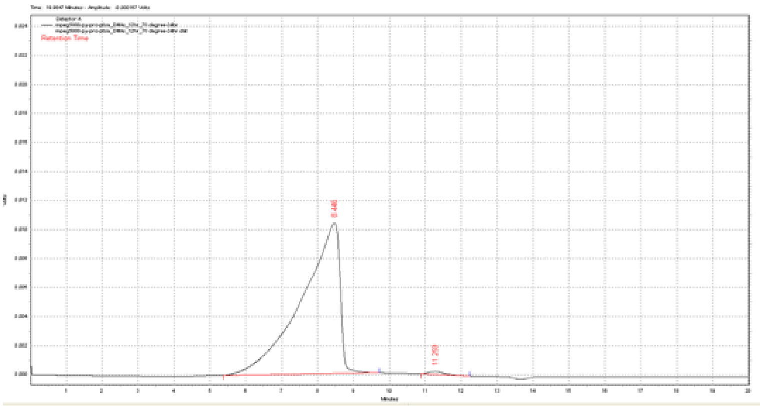

(c)

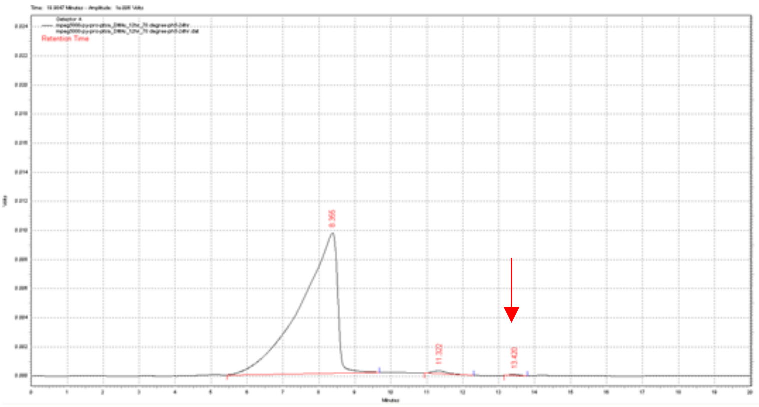

Figure S3. GPC spectrum of the mPEG-b-P(HEMA-ketal-VB_6) in various pH conditions. (a) The GPC spectrum of mPEG-b-P(HEMA-ketal-VB_6) before any treatment. (b) The GPC spectrum of mPEG-b-P(HEMA-ketal-VB_6) in pH 7.4 conditions for 24 h incubation. (c) The GPC spectrum of mPEG-b-P(HEMA-ketal-VB6) in pH 5.0 conditions for 24 h incubation.

S-4. Preparation and characterization of mPEG-b-P(MAAc) copolymer

On the basis of the integral areas of the methylene groups in MAAc (δ 1.6-2.0) and the methylene groups in mPEG (δ 3.5), there were approximately 33 repeating units for the copolymers, as **Figure S4a** shows. The FT-IR spectrum in **Figure S4b** presents peaks representing -COO- and -OH stretching at the wavenumbers of 1572 and 2882 cm^{-1}. The ^{1}H-NMR and FT-IR spectral analysis identified the successful synthesis of mPEG-b-PMAAc.

Figure S4.

(a)

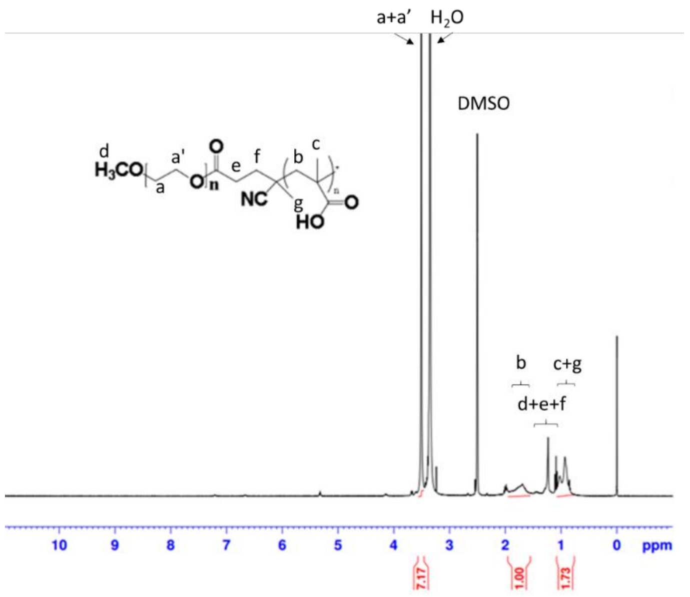

(b)

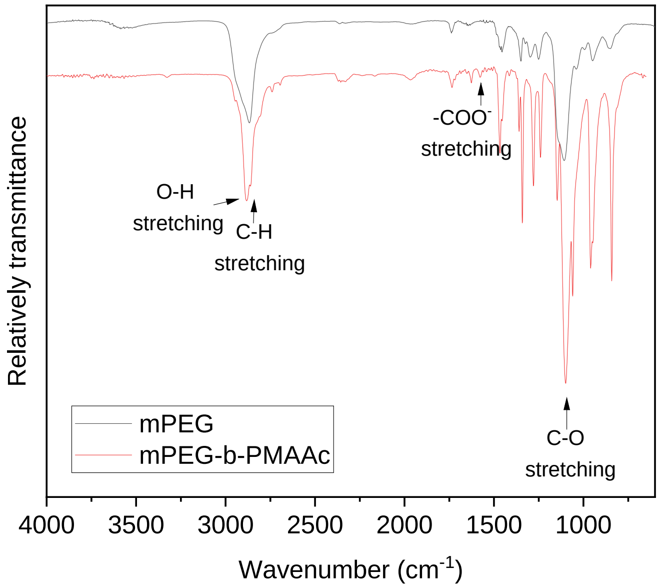

Figure S4. Preparation and characterization of the mPEG-b-P(MAAc) copolymers. (a) The ^{1}H-NMR spectrum of the mPEG-b-P(MAAc) copolymers in DMSO-d_6. (b) The FT-IR spectrum of the mPEG-b-P(MAAc) copolymers.

S-5. Modification and characterization of mPEG-b-P(MAAc-co-NHS) copolymer

For the ^{1}H-NMR spectrum shown in **Figure S5a** in **Supporting Information**, the chemical shift of methylene groups in NHS ester appeared at approximately 2.7 ppm. On the basis of its ratio of the integral areas with the methylene groups of MAAc, the conjugation rate was 71%. The FT-IR spectrum in **Figure S5b** displays N-O and C=O stretching peaks, identifying the successful conjugation and synthesis of mPEG-b-

P(MAAc-co-NHS ester).

Figure S5.

(a)

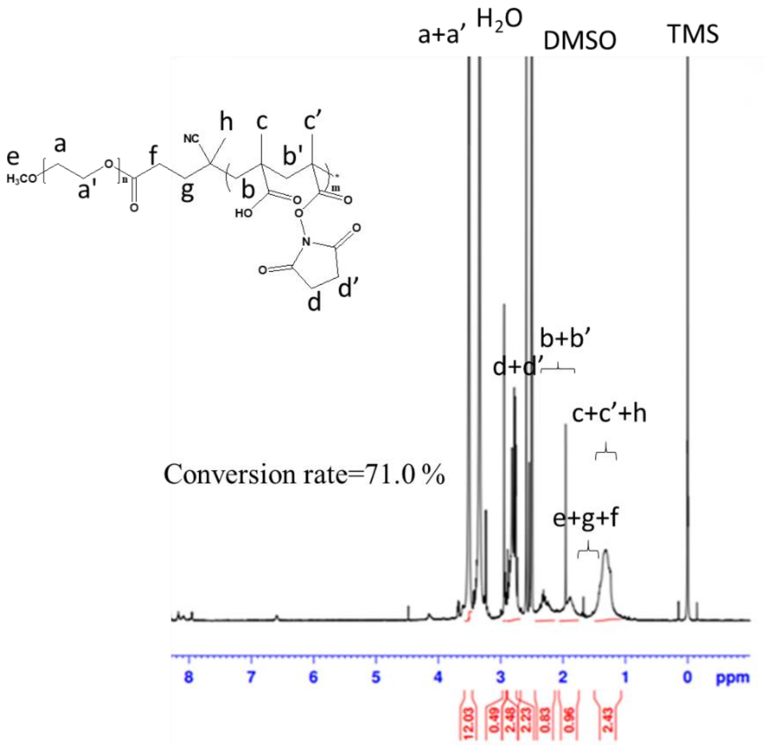

(b)

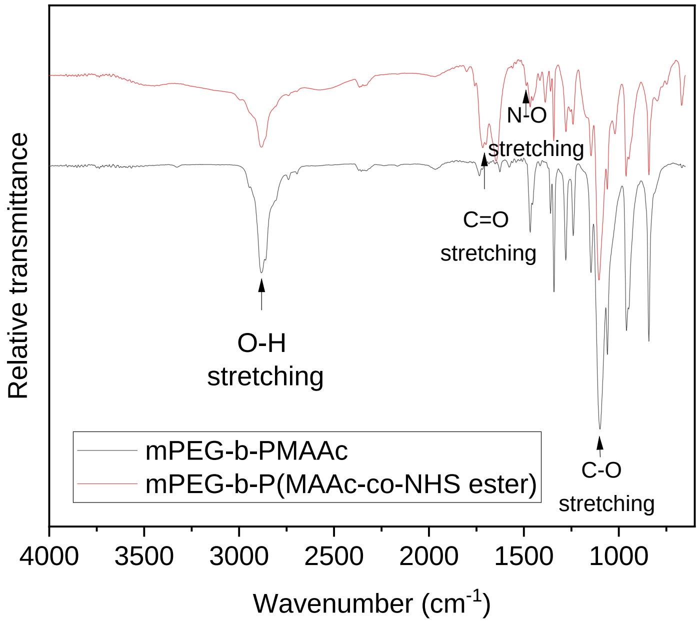

Figure S5. Modification and characterization of the mPEG-b-P(MAAc-co-NHS)

copolymers. (a) The ^{1}H-NMR spectrum of the mPEG-b-P(MAAc-co-NHS) copolymers in DMSO-d_6. (b) The FT-IR spectrum of the mPEG-b-P(MAAc-co-NHS) copolymers.

S-6. *Preparation and characterization of the polyplex*

mPEG-b-P(HEMA-ketal-VB_6) has positively charged pyridine groups which is its ability to carry anionic siRNA and form the polyplex was examined. As first, the ability of the copolymer to attract the siRNA and optimum incubation periods were evaluated. Hence, various molar concentrations of the mPEG-b-P(HEMA-ketal-VB_6) and the siRNA (N/P ratio) were co-incubated to form the polyplexes. At 3-4 h, 8-9 h, and 24 h post-incubation, the polyplexes were identified using gel electrophoresis. In **Figure S6a**, where the bands represent free siRNA, the red arrow indicates that they could be observed when the polymer was incubated with siRNA for 3-4 h and 8-9 h. However, the band vanished when the polymer and siRNA were co-incubated at a ratio of 100:1 over 8 h. The results suggest that the polymers and siRNA should be incubated for at least 8-9 h with the ratio of 100:1 such that they could completely form the desired complexes.

Furthermore, the attraction between the siRNA and the copolymer mPEG-b-P(HEMA-ketal-VB_6) was also detected in the UV-vis spectrum. As **Figure S6b** shows, vitamin B_6 displayed absorbance at 302.1 nm, while after conjugation onto the polymers the peaks subtly shifted to 305.2 nm. When the polymers were incubated with siRNA under the ratio of 100:1, the absorbance peak at 305.2 nm still could be detected. It is noticeable that an absorbance peak at 285.1 nm appeared as the siRNA and polymers were co-cultured. With the incubation period of 24 h, when the free siRNA could not be detected via gel electrophoresis, the absorbance at 285.1 nm increased. The peak at 285.1 nm could be ascribed to the polyplex formation. The electrical attractions between siRNA and the polymers influenced the distance of molecules inside the polyplexes. With the increasing incubation period, the inner structures of the polyplexes tightly package and meanwhile, more siRNA interacted with the polymers, hence the absorbance peaks at 285.1 nm increased.

The polyplex structure was further observed using transmission electron microscopy (TEM) after staining with uranyl acetate. The TEM images, shown in **Figure S6c**, display the tightly packaged polyplex structures and simultaneously the PEG shells could be also witnessed around the polyplexes. When the tight polyplexes were incubated in pH 5.0 conditions for 24 h, the architectures of the polyplexes deformed as shown in **Figure S6d**. The deformation was caused by the cleavage of

the ketal bonds. This further identified that the pH responsiveness of the polymer was preserved after forming the tightly packaged polyplex; however, the TEM images in **Figure S6c** clearly indicate that the particle sizes of the polyplexes were over 1 μm in diameter. The measurement of the dynamic light scattering further identified that their hydrodynamic diameters of the polyplexes were 4750 nm.

(a)

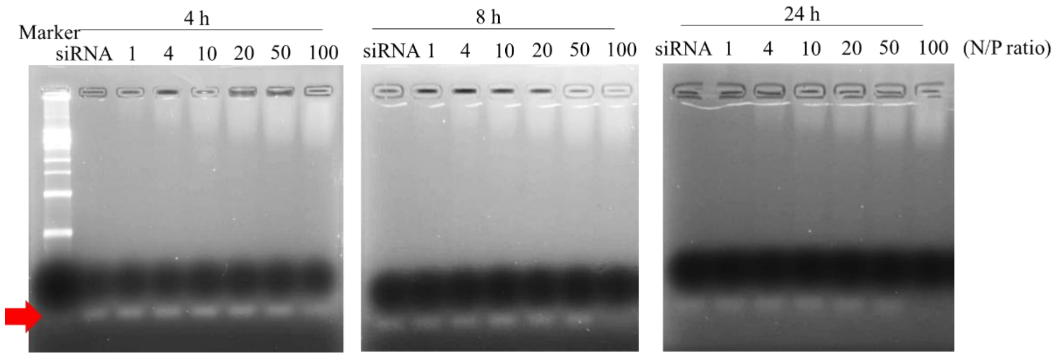

(b)

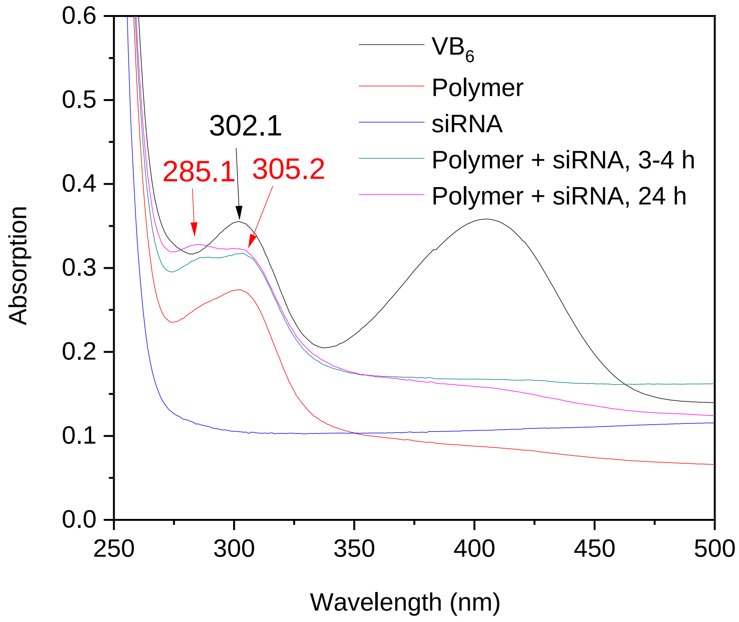

(c) (d)

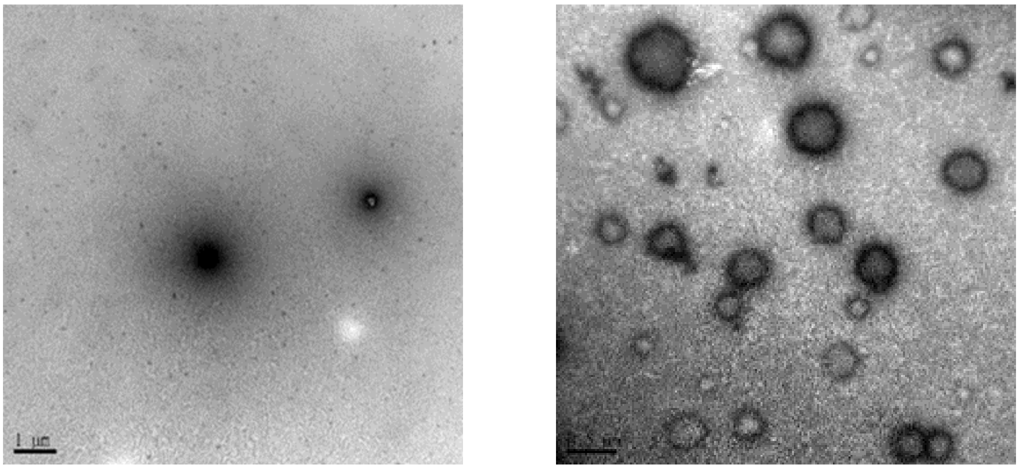

Figure S6. Preparation and characterization of the polyplexes. Various concentrations of the copolymer, mPEG-b-P(HEMA-ketal-VB_6), were independently incubated with the FAK siRNA for 3-4 h, 8-9 h, and 24 h to form the polyplexes. After different incubation periods, the polyplexes were analyzed with (a) gel electrophoresis and (b) UV-vis spectrum analysis. (c) Transmission electron microscopy (TEM) images of the polyplexes. (d) The TEM images of the polyplexes after incubation in pH 5.0 for 24 h. The morphology of the polyplexes were both observed using TEM after staining with 2% uranyl acetate.
